# Serum Urate and Renal Dysfunction: Bidirectional Genetic Associations, External Validation, and Exploratory Multi-Omics Characterization of Antithrombin (SERPINC1) in Hyperuricemic Nephropathy

**DOI:** 10.3390/genes17091084

**Published:** 2026-09-09

**Authors:** Lei Jin, Feng Wang

**Affiliations:** Department of Rheumatology, Immunology & Allergy, Shanghai General Hospital, Shanghai Jiao Tong University School of Medicine, Shanghai 200080, China; leijin1987@hotmail.com

**Keywords:** antithrombin, SERPINC1, nephropathy, hyperuricemia, thromboinflammation

## Abstract

**Background:** Hyperuricemic nephropathy (UAN) involves a systemic thromboinflammatory axis, yet the role of antithrombin (AT, SERPINC1) remains incompletely defined. While AT has anticoagulant and anti-inflammatory properties, whether it contributes to UAN as a disease-responsive molecular node remains uncertain. **Methods:** We integrated two-sample MR of serum urate and renal traits with complementary sensitivity analyses, cis-eQTL and pQTL analyses of SERPINC1, and exploratory transcriptomic, proteomic, metabolomic, and coexpression analyses. The available data were used to assess genetic associations and to distinguish genetic evidence from cross-dataset molecular patterns. **Results:** Genetically predicted serum urate showed positive or negative associations with BUN and eGFR, respectively, but the primary analyses showed substantial heterogeneity. Reverse-direction estimates were also associated with serum urate, representing reciprocal genetic relationships between related renal traits and urate rather than proof of a temporal feedback cycle. Available data did not demonstrate mediation through genetically predicted whole-blood SERPINC1 expression. Three deCODE cis-pQTL instruments gave inconsistent renal estimates: nominally lower BUN (β = −0.0290, *p* = 0.047), null eGFR (β = −0.0036, *p* = 0.472), and higher CKD risk (β = +0.3361, *p* = 0.020) with directional pleiotropy evidence. Exploratory multi-omics analyses showed higher PBMC SERPINC1 expression during acute gout and a nominal urinary proteomic difference that did not survive multiple-testing correction (FDR = 0.998). **Conclusions:** SERPINC1 is best interpreted as a responsive molecular feature requiring further validation, rather than as a demonstrated genetic mediator or renal-protective therapeutic target. The transcriptomic and urinary findings represent cross-dataset, stage-associated observations and should not be interpreted as proven sequential phases.

## 1. Introduction

Hyperuricemic nephropathy (UAN) is a significant chronic kidney disease (CKD) caused by purine metabolism abnormalities. Before urate-lowering therapy, up to 25% of gout patients have renal damage, and 20–40% show reduced function; autopsy findings confirm widespread renal pathology in gout [1]. While traditional mechanisms focused on urate crystal deposition, current evidence highlights a systemic ‘thrombotic–inflammatory’ network triggered by soluble urate and crystals as key to disease progression [2]. Epidemiological studies link elevated uric acid to increased thrombotic risk [3], with hyperuricemia and gout being independent risk factors for venous thromboembolism, atrial thrombi, and atherosclerotic events [4]. Targeting coagulation, particularly antithrombin (AT) and its anticoagulant–anti-inflammatory effects, is thus a promising intervention.

AT, encoded by SERPINC1, shows organ-protective effects beyond anticoagulation in CKD [5,6,7,8]. The gene, located on chromosome 1, encodes 464 amino acids [9]. AT inhibits serine proteases in coagulation and exerts anti-inflammatory activity partly via prostacyclin. SERPINC1 variants can increase thromboembolism risk [10]. The eExisting literature on SERPINC1 and kidney disease focuses on AT deficiency-related injury, with limited genetic or molecular insights in hyperuricemic kidney damage.

Therefore, we hypothesized that AT (SERPINC1) might link hyperuricemia, systemic thromboinflammation, and renal injury. To examine this hypothesis, we integrated Mendelian randomization (MR), GEO transcriptomics, Weighted Gene Co-expression Network Analysis (WGCNA), urinary proteomics, plasma/urine metabolomics, cis-expression quantitative trait locus (eQTL) analysis, and circulating-protein pQTL analysis. The analyses were designed to distinguish genetic associations from exploratory molecular patterns across different tissues, cohorts, and experimental platforms. We also assessed the urate–kidney relationship in FinnGen R12 as external validation and phenotypic extension, rather than assuming one-to-one replication of all CKDGen outcomes.

## 2. Methods

### 2.1. Overview of Study Design and Data Sources

This study employed a multi-stage, multi-omics integrative design. The analytical workflow, as illustrated in Supplementary Figure S1, comprised: (1) two-sample and bidirectional MR to assess reciprocal genetic relationships between serum urate and renal traits (BUN, eGFR, and CKD); (2) cis-eQTL MR and a planned two-step mediation framework, subject to first-step feasibility, to assess SERPINC1; (3) exploratory transcriptomic, proteomic, and metabolomic characterization; (4) sensitivity analyses to assess heterogeneity and pleiotropy; and (5) PPI and functional analyses to summarize molecular context. Details of each module are provided below.

This study employs a multi-omics integrated analysis strategy to systematically investigate the genetic associations and molecular mechanisms of AT (SERPINC1) in UAN. The analysis encompasses four dimensions: genetic, transcriptomic, proteomic, and metabolomic. All data are derived from published, publicly available large-scale databases or datasets; no new patient samples were collected, and, therefore, no additional ethical approval was required. Detailed information on each dataset is summarized in Table 1.

### 2.2. Mendelian Randomization Analysis

To investigate the genetic relationship between serum urate and renal traits, we used a two-sample MR framework. The primary analysis estimated the forward association of urate with renal outcomes, while complementary analyses assessed sensitivity to heterogeneity, pleiotropy, and reverse-direction genetic relationships. The results were interpreted as genetic associations with calibrated causal certainty rather than as proof of a temporal biological feedback loop.

#### 2.2.1. Instrumental Variable Selection

Instrumental variables (IVs) for serum uric acid were derived from a European GWAS meta-analysis (GWAS Catalog ID: GCST90319904). Single-nucleotide polymorphisms (SNPs) significantly associated with uric acid levels at the genome-wide significance level (*p* < 5 × 10^−8^) were selected. These were subsequently clumped for independence (linkage disequilibrium r^2^ < 0.001 within a 10,000 kb window), yielding 351 independent genetic variants. The F-statistic for all IVs exceeded 10, with a mean of 153.0, indicating no weak instrument bias. To satisfy the exclusion restriction assumption, we used the PhenoScanner database to identify and exclude pleiotropic IVs associated (*p* < 5 × 10^−8^) with potential confounders of renal function, such as body mass index, diabetes, blood pressure, and blood lipids. The final number of IVs used for each outcome was 331 for BUN, 341 for eGFR, and 343 for CKD.

Data for renal function outcomes were sourced from a large-scale meta-analysis by the CKDGen Consortium, including BUN (N > 200,000), eGFR (N > 300,000), and CKD (total cases = 41,395).

Instrumental variables for SERPINC1 gene expression were cis-expression quantitative trait loci (cis-eQTLs) obtained from the eQTLGen Phase 1 database [16] (whole blood, N = 31,684). Independent cis-eQTLs within 1 Mb upstream/downstream of SERPINC1 were selected after pruning, resulting in 7 IVs for summary-data-based MR. Because the available expression data are from whole blood, the analysis cannot exclude tissue-specific regulation in the kidney. Details of the IVs are provided in Table 2.

#### 2.2.2. Primary, Sensitivity, and Extended Analyses

Causal effect estimates were derived using fixed-effect inverse-variance weighted (IVW) MR as the conventional primary estimator. When between-instrument heterogeneity was substantial, multiplicative random-effects IVW estimates were reported alongside the fixed-effect estimates. Sensitivity analyses included MR-Egger regression, weighted median, weighted mode, and MR-PRESSO. Heterogeneity was assessed using Cochran’s Q test, and the Steiger test was used to assess the assumed direction. MR-PRESSO outlier removal was treated as sensitivity evidence and does not eliminate residual pleiotropy.

Extended analyses comprised: (1) bidirectional MR, with renal traits as exposures and serum urate as the outcome, to assess reciprocal genetic relationships; (2) a planned two-step MR mediation analysis, which was reported only if the first urate-to-SERPINC1 expression step was adequately supported; and (3) Bayesian colocalization to assess compatibility of SERPINC1 eQTL and renal-trait signals at the locus. The absence of a supported first step was not treated as proof that SERPINC1 cannot mediate disease through other tissues or non-genetic pathways.

#### 2.2.3. Statistical Analysis and Software

All MR analyses were performed in R (version 4.3.2) using the TwoSampleMR (v0.5.7), MRPRESSO (v1.0), and coloc (v5.2.1) packages. Statistical significance was defined as a two-sided *p*-value < 0.05, with multiple-testing thresholds stated where applicable. Cochran’s Q *p* < 0.05 indicated heterogeneity, MR-Egger intercept *p* < 0.05 suggested directional pleiotropy, and the Steiger test assessed the assumed direction.

Transcriptomic differential expression was assessed using Welch’s *t*-test, and the exploratory coexpression analysis used the locally documented WGCNA workflow. Proteomic differential analysis employed the Wilcoxon rank-sum test, with multiple-testing correction via the Benjamini–Hochberg method. Metabolic pathway enrichment was assessed using Fisher’s exact test. Figures were generated using R (version 4.2.1) with ggplot2 (version 3.4.2) and Python (version 3.9.7) with Matplotlib (version 3.5.3). The complete analytical workflow is documented in the project materials; exploratory network and cross-dataset findings are interpreted with their sample-size and tissue limitations.

### 2.3. Clinical Implications and Translational Potential

This study was designed to characterize the genetic and molecular relationships linking serum urate, renal traits, and SERPINC1, rather than to establish an actionable treatment framework. We aimed to (1) estimate the forward and reverse genetic relationships between urate and renal outcomes, (2) assess whether available SERPINC1 eQTL and pQTL data support a genetic mediator or protein-level association, and (3) summarize exploratory transcriptomic, proteomic, metabolomic, and PPI evidence. FinnGen R12 was used for external validation and phenotypic extension. Urinary antithrombin and PBMC SERPINC1 expression may be useful hypotheses for future biomarker studies, but neither the current data nor the pQTL results establish a clinical biomarker or therapeutic benefit.

### 2.4. SERPINC1 pQTL MR and External Validation in FinnGen

#### 2.4.1. SERPINC1 pQTL Instrumental Variables

To assess associations of circulating antithrombin protein levels with renal outcomes, we obtained SERPINC1 cis-pQTL summary statistics from the deCODE plasma proteome GWAS (N = 35,559, Ferkingstad et al. [17]). Four candidate cis-pQTL variants met the local screening criteria. Three variants available across all three CKDGen outcomes were used in the main analyses: rs116185683, rs141846315, and rs112808303. The additional candidate rs2016525 was not available across all three outcome datasets and was not included in the common three-instrument analyses. This was a relaxed cis-instrument analysis rather than a set in which every variant reached *p* < 5 × 10^−8^.

For external validation and phenome-wide exploration, we additionally used SERPINC1 pQTL instruments from the UK Biobank Pharma Proteomics Project (UKB PPP Olink 3k, Sun et al. [18]). Twenty genome-wide significant pQTL variants, including four cis variants, were reported in the submitted analysis and evaluated across 624 FinnGen R12 endpoints (Kurki et al. [19]). The count of 624 is used consistently because it matches the submitted results, the supplementary table, and the stated Bonferroni threshold of approximately 0.05/624 = 8.0 × 10^−5^ (Kurki et al. [19]).

#### 2.4.2. pQTL Mendelian Randomization and Colocalization

MR analysis was performed using the framework described above. For the deCODE pQTL instruments, IVW was used as the primary method, with MR-Egger, weighted median, and weighted mode analyses used for sensitivity assessment. With only three common instruments, these estimates were interpreted cautiously. For the 624-endpoint FinnGen PheWAS, Bonferroni correction (*p* < 8.0 × 10^−5^) and FDR correction (q < 0.05) were applied. Bayesian colocalization assessed whether pQTL and disease signals were compatible with a shared causal variant; it was not interpreted as proof of kidney-specific causality.

#### 2.4.3. External Validation of the Urate-Kidney Genetic Relationship

The original 351 serum urate GWAS instruments were mapped to 11 pre-defined kidney endpoints in FinnGen R12. IVW, weighted median, and MR-Egger analyses were used to assess external validation of the urate–kidney genetic relationship. These analyses were treated as phenotypic extension rather than one-to-one replication of every CKDGen outcome.

### 2.5. Transcriptomic Data (GSE160170) and Exploratory Coexpression Analysis

To investigate changes in SERPINC1 expression under disease conditions, we analyzed transcriptomic datasets from the NCBI GEO database. The core discovery dataset, GSE160170, was generated on GPL21827 and comprises PBMC samples from 12 Chinese males [13]: acute gout (*n* = 3), intercritical gout (*n* = 3), and healthy controls (*n* = 6). Differential expression and an exploratory coexpression workflow were applied to this small dataset. The selected soft-threshold power was β = 13, with scale-free topology R^2^ = 0.807 in the reproducible local output. Multiple peripheral-blood and renal-tissue GEO datasets were summarized in a cross-dataset meta-analysis.

### 2.6. Proteomics Data (PXD016900)

To validate the findings at the protein level, we analyzed a urinary proteomics dataset from ProteomeXchange. Dataset PXD016900 comprises urine samples from 51 Chinese individuals, including 26 patients with hyperuricemia and 25 healthy controls. The data were acquired using a liquid chromatography–tandem mass spectrometry (LC-MS/MS) platform and processed using MaxQuant, resulting in the quantification of 1577 proteins. This dataset was used to identify urine proteins differentially expressed in the hyperuricemia condition, and the quantitative values for SERPINC1 were extracted for specific analysis.

### 2.7. Metabolomics Data

To investigate associated metabolic pathway dysregulation, we utilized metabolomics data from published studies. The metabolite data were sourced from the Supplementary Materials of a publicly published paper [15]. This study compared plasma and urine metabolomic profiles between patients with gout and those with asymptomatic hyperuricemia. Based on the list of differentially expressed metabolites reported therein, we performed Kyoto Encyclopedia of Genes and Genomes pathway enrichment analysis to identify metabolic pathway alterations associated with hyperuricemic kidney injury.

### 2.8. Descriptive Multi-Omics Evidence Summary

Evidence from MR, transcriptomics, exploratory coexpression analysis, proteomics, metabolomics, and PPI analysis was summarized across six descriptive dimensions. No composite score, percentage, independent validation claim, or all-gene ranking is reported because the original scoring rule was not independently validated and several dimensions are statistically or biologically non-independent.

## 3. Research Results

### 3.1. Genetic Associations of Serum Urate with Renal Function

**Figure 1:**
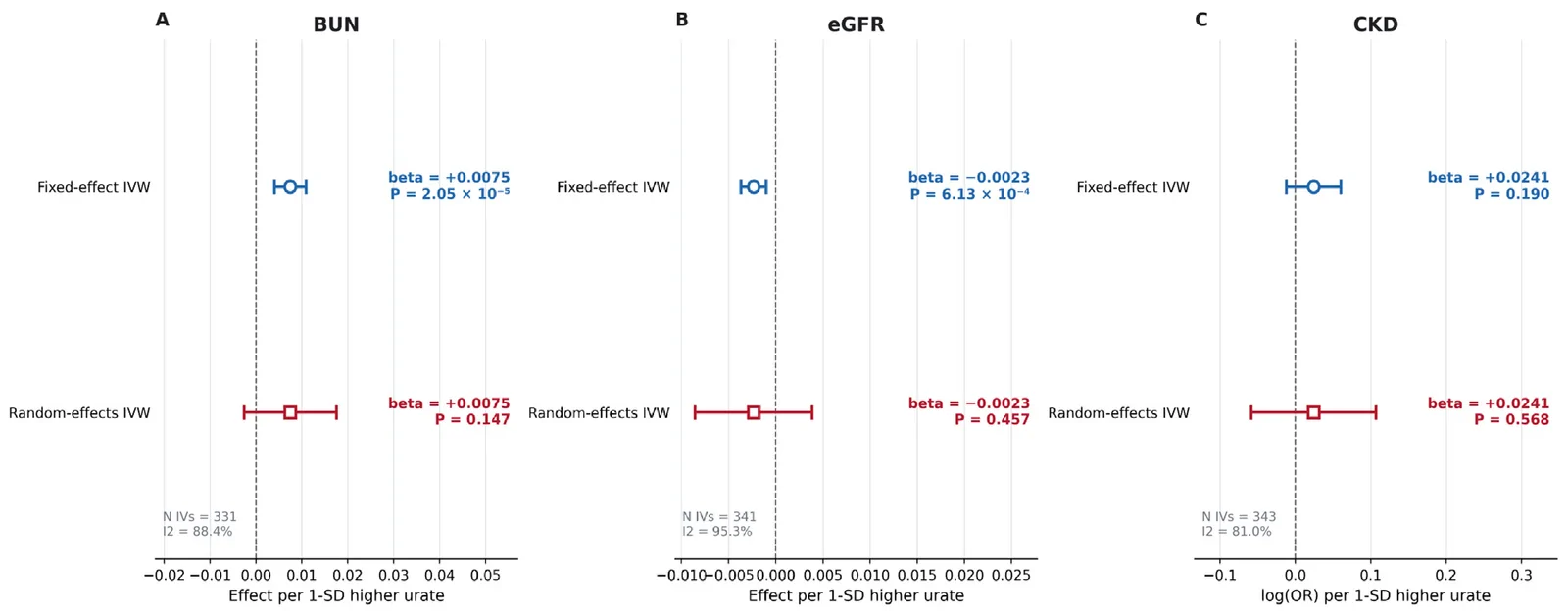
Serum urate and kidney outcomes: primary and heterogeneity-adjusted MR estimates. (**A**) BUN, (**B**) eGFR, (**C**) CKD. Each panel shows fixed-effect and multiplicative random-effects IVW estimates with 95% confidence intervals. The number of instrumental variables and I^2^ are shown for each outcome. Abbreviations: BUN, blood urea nitrogen; eGFR, estimated glomerular filtration rate; CKD, chronic kidney disease; IVW, inverse-variance weighted; MR, Mendelian randomization.

To establish the genetic relationship between serum urate and renal traits, we performed two-sample MR using 351 independent IVs for serum urate (mean F-statistic = 153.0, all >29) (Table 1). Fixed-effect IVW showed higher urate associated with higher BUN (β = +0.00745, SE = 0.00175, *p* = 2.05 × 10^−5^) and lower eGFR (β = −0.00235, SE = 0.00069, *p* = 6.13 × 10^−4^). The CKD estimate was positive but not statistically significant (β = +0.02410, *p* = 0.190) (Figure 1A, Table 2). Given the high heterogeneity, the multiplicative random-effects IVW estimates were not statistically significant for BUN (*p* = 0.147), eGFR (*p* = 0.457), or CKD (*p* = 0.568).

Sensitivity analyses showed high heterogeneity for BUN, eGFR, and CKD (I2 = 88.4%, 95.3%, and 81.0%, respectively). MR-PRESSO outlier removal retained the direction of the estimates and produced *p* values of 3.56 × 10^−6^, 1.80 × 10^−5^, and 0.054 for BUN, eGFR, and CKD, respectively; these are sensitivity results and do not remove residual pleiotropy. Leave-one-out analyses retained the direction of the full IVW estimate: BUN β ranged from +0.00538 to +0.01002, eGFR from −0.00348 to −0.00158, and CKD from +0.01384 to +0.04284. No single instrument was identified as solely determining the direction by this diagnostic. The Steiger test supported the assumed direction for 97.6–99.7% of IVs (Supplementary Figure S2).

### 3.2. Bidirectional MR Shows Reciprocal Genetic Relationships

To assess reverse-direction genetic relationships, we performed bidirectional MR. Renal traits were associated with serum urate: BUN increased serum urate (β = +1.227, *p* = 3.89 × 10^−181^), lower eGFR was associated with higher urate (β = −0.802, *p* = 5.76 × 10^−35^), and CKD showed a positive association with urate (β = +0.189, *p* = 5.56 × 10^−152^) (Table 3). These estimates are compatible with reciprocal genetic relationships, but BUN, eGFR, and CKD use different trait-specific scales and are not directly comparable in magnitude. They do not by themselves establish a temporal biological feedback process.

### 3.3. SERPINC1 Genetic Mediation Was Not Demonstrated

We next assessed whether the available SERPINC1 genetic data support mediation of the urate–kidney relationship. The results were interpreted as evidence about the available whole-blood eQTL framework, not as proof that SERPINC1 cannot mediate disease through other tissues or non-genetic pathways.

**Figure 2:**
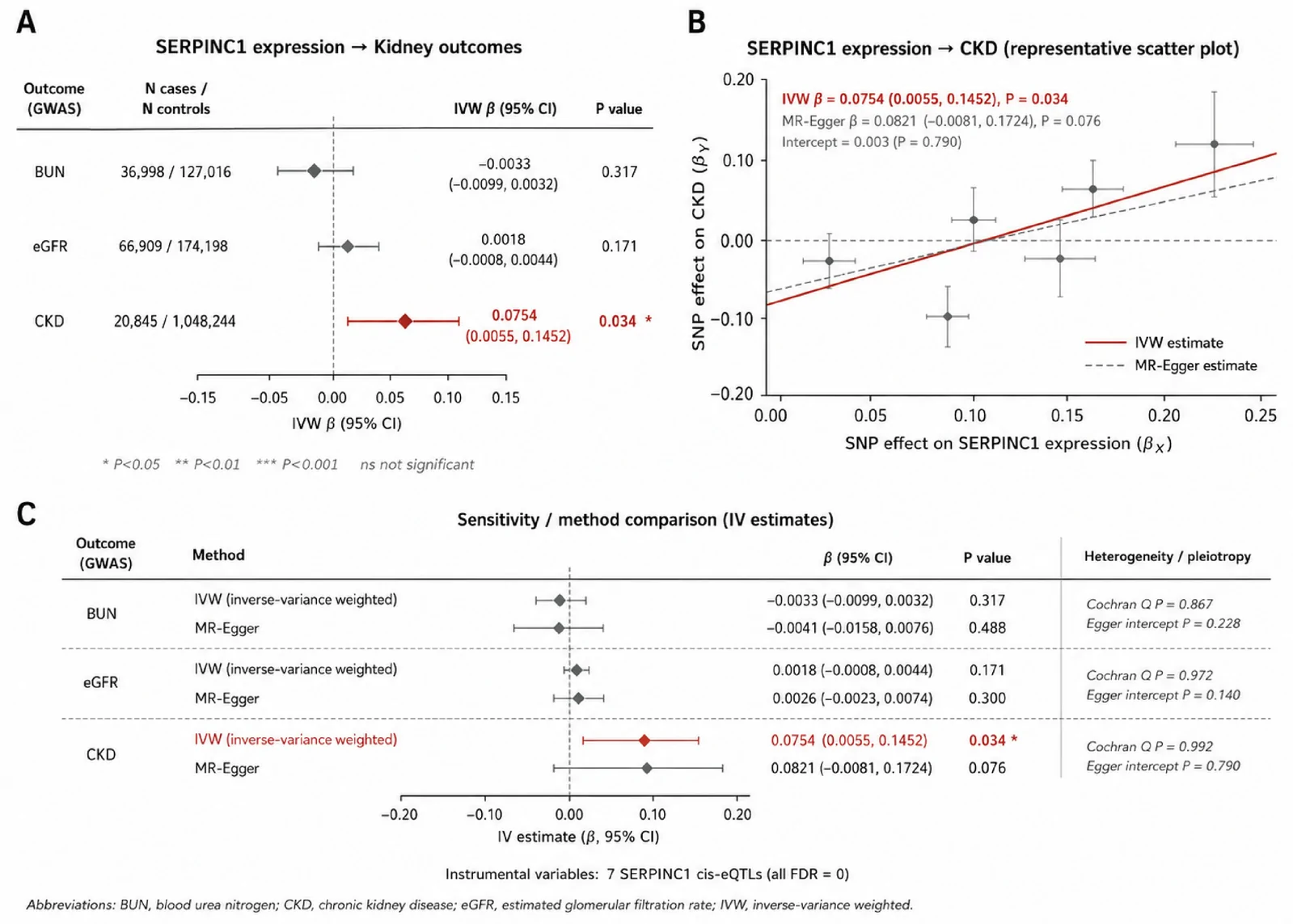
Whole-blood SERPINC1 cis-eQTL MR analysis. (**A**) Forest plot showing associations of genetically predicted SERPINC1 expression with BUN, eGFR, and CKD using seven cis-eQTL instruments. (**B**) Representative scatter plot for the SERPINC1 expression–CKD association; the solid red line represents the IVW estimate and the dashed gray line represents the MR-Egger estimate. (**C**) Sensitivity and method-comparison estimates for BUN, eGFR, and CKD using IVW and MR-Egger methods. Asterisks denote nominal significance levels (* *p* < 0.05). Abbreviations: BUN, blood urea nitrogen; CKD, chronic kidney disease; eGFR, estimated glomerular filtration rate; eQTL, expression quantitative trait locus; IVW, inverse-variance weighted; MR, Mendelian randomization.

Cis-eQTL MR analysis: using seven SERPINC1 expression IVs from eQTLGen (all FDR = 0) (Table 4) showed no significant association of genetically predicted SERPINC1 expression with BUN (β = −0.00334, *p* = 0.317) or eGFR (β = +0.00180, *p* = 0.171), with a nominal association for CKD (β = +0.07537, *p* = 0.034) (Figure 2B, Table 5). Sensitivity analyses were interpreted cautiously given the limited number of instruments (Supplementary Figure S3).

Two-step MR mediation analysis: The planned two-step MR mediation analysis was not estimable because no sufficiently supported first-step association between genetically predicted urate and whole-blood SERPINC1 expression was available in the current package. This prevents a valid two-step mediation estimate; it does not exclude relationships between urate and SERPINC1 in other tissues or through non-genetic pathways.

Bayesian colocalization analysis: Bayesian colocalization was used to compare the SERPINC1 eQTL signal with renal function GWAS signals in the locus. The available results did not provide supportive evidence for a shared causal signal. Because colocalization depends on tissue, variant coverage, and model assumptions, this result is not interpreted as proof that no shared causal variant exists in another tissue or dataset.

**Figure 3:**
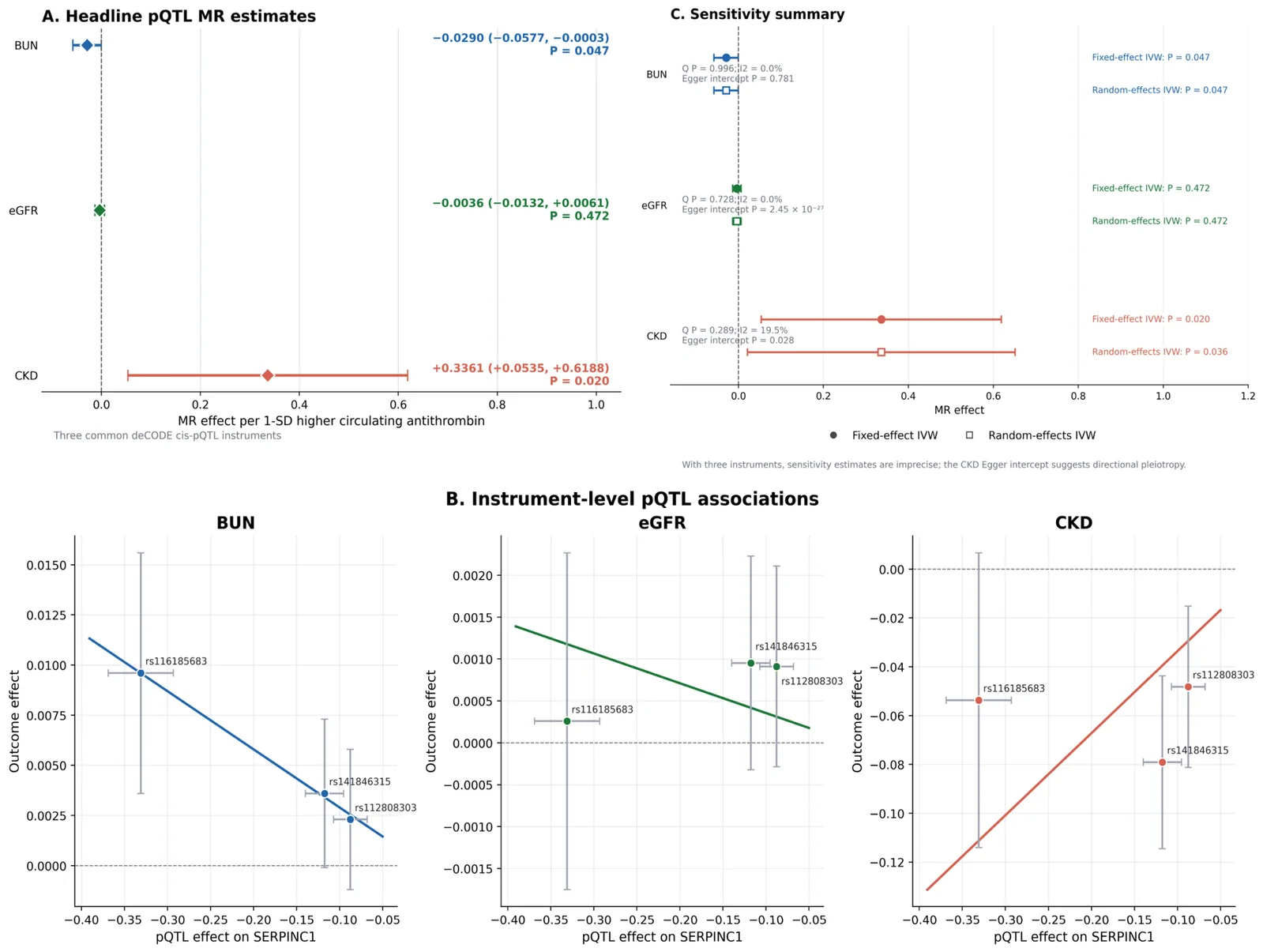
Circulating SERPINC1 protein pQTL MR analysis. (**A**) Forest plot showing associations of genetically predicted circulating antithrombin protein levels with BUN, eGFR, and CKD using the three common deCODE cis-pQTL instruments rs116185683, rs141846315, and rs112808303; diamonds show IVW point estimates with 95% confidence intervals. (**B**) Instrument-level scatter plots across the three kidney outcomes; labeled points identify the three instruments. (**C**) Sensitivity analyses; filled circles denote fixed-effect IVW estimates and open squares denote multiplicative random-effects IVW estimates. These findings are preliminary and do not establish a general renal-protective effect. Abbreviations: pQTL, protein quantitative trait locus; IVW, inverse-variance weighted.

Complementary pQTL MR analysis: Using three common deCODE cis-pQTL IVs (mean F = 36.1) (Supplementary Table S1) showed a nominal association with lower BUN (β = −0.0290, *p* = 0.047), a null association with eGFR (β = −0.0036, *p* = 0.472), and a higher CKD estimate (β = +0.3361, *p* = 0.020) accompanied by directional pleiotropy evidence (MR-Egger intercept *p* = 0.028) (Figure 3A, Table 6). These three-instrument results are preliminary and inconsistent; they do not establish a general protective effect or therapeutic benefit.

Taken together, the available analyses did not demonstrate mediation through genetically predicted whole-blood SERPINC1 expression. This should not be interpreted as evidence of biological irrelevance. Whole-blood eQTLs may not capture kidney-specific regulation, and transcriptional, circulating-protein, and urinary measurements represent distinct biological layers. The pQTL estimates are preliminary and inconsistent, so they do not establish a therapeutic or biomarker effect.

### 3.4. Multi-Omics Characterization of SERPINC1-Associated Patterns

With the genetic role clarified, we next characterized the molecular behavior of SERPINC1 across disease stages using transcriptomic, proteomic, and metabolomic data.

**Figure 4:**
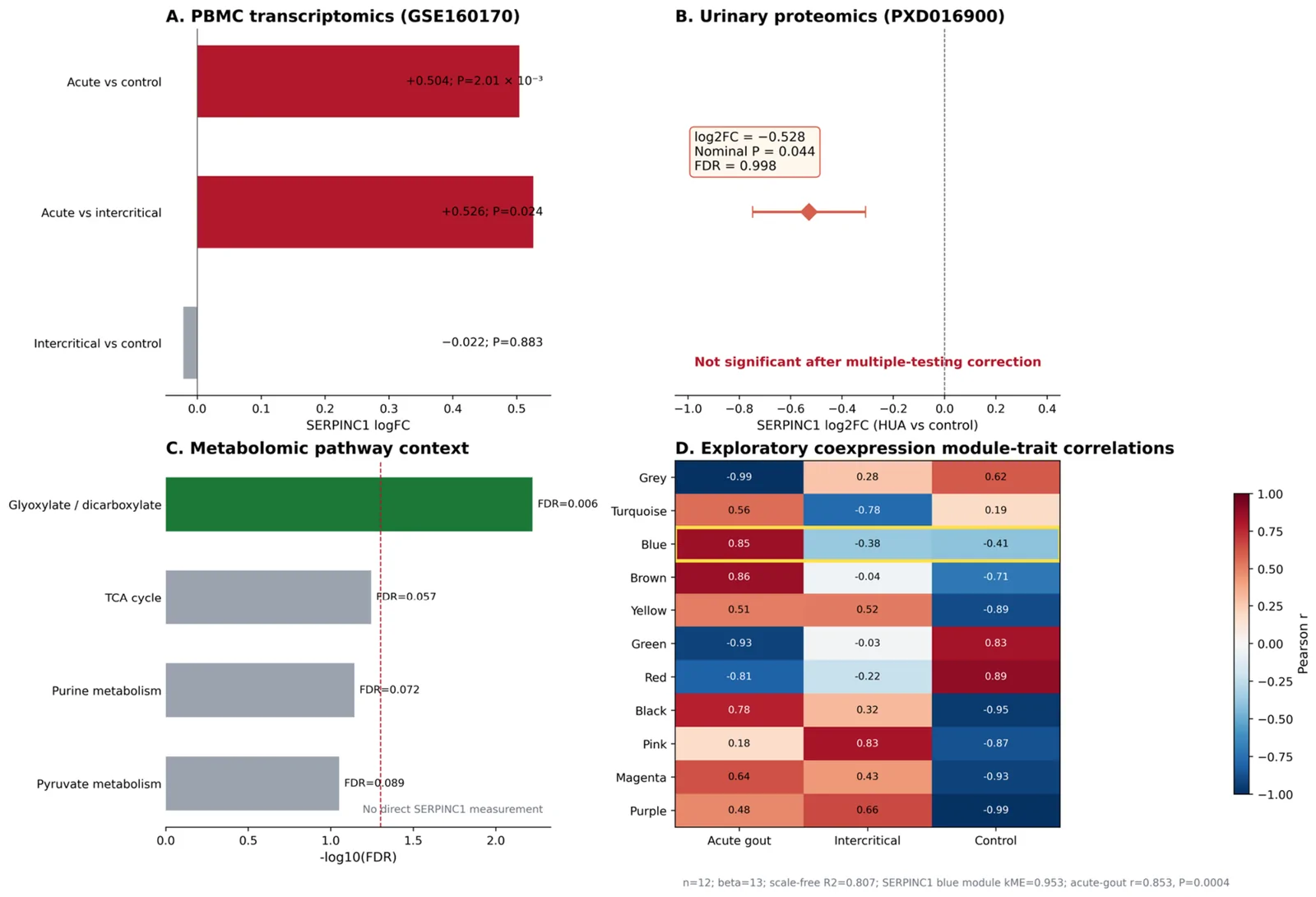
Multi-omics molecular characterization of SERPINC1-associated patterns. (**A**) Transcriptomic analysis of SERPINC1 expression in PBMCs across disease stages (GSE160170); red bars denote nominally significant acute-gout comparisons and the gray bar denotes a non-significant comparison. (**B**) Urinary proteomic analysis (PXD016900); the coral diamond and interval show the nominal SERPINC1 estimate and 95% confidence interval, which did not survive FDR correction. (**C**) Metabolomic pathway context (PMC10887286); green denotes FDR < 0.05 and gray denotes pathways that did not meet FDR < 0.05. (**D**) Exploratory WGCNA module-trait correlation heatmap; red and blue indicate positive and negative Pearson correlations, respectively, and the yellow outline highlights the blue module. Abbreviations: PBMC, peripheral blood mononuclear cell; FDR, false discovery rate; WGCNA, weighted gene co-expression network analysis.

Transcriptomic analysis of the GSE160170 dataset (human PBMCs, *n* = 12) showed higher SERPINC1 expression in acute gout than in healthy controls (logFC = +0.504, *p* = 0.002) and the intercritical phase (logFC = +0.526, *p* = 0.024), with no difference between intercritical and control groups (*p* = 0.883) (Figure 4A). In exploratory coexpression analysis, the selected soft-threshold power was β = 13 (scale-free topology R^2^ = 0.807). SERPINC1 had high intramodular connectivity in the blue module (kME = 0.953), which correlated with acute gout (r = 0.853, *p* = 0.0004). Given the total sample size of 12 and three samples in each gout subgroup, these findings do not establish a stable or generalizable core-hub designation. Cross-dataset meta-analysis showed peripheral-blood upregulation (pooled logFC = +0.736, *p* = 1.58 × 10^−4^, I^2^ = 0%) but no differential expression in renal tissue (pooled logFC = −0.059, *p* = 0.496) (Supplementary Figure S4).

Proteomic analysis of PXD016900 (urinary proteome, *n* = 51) showed a nominal SERPINC1 difference in hyperuricemia versus controls (log_2_FC = −0.528, *p* = 0.044, FDR = 0.998) (Figure 4B). Because the result did not survive multiple-testing correction, it should not be interpreted as strong evidence of consumptive depletion. PPI analysis identified SERPINC1 as a highly connected node (degree = 50) in the constructed network. The six-dimension evidence display is descriptive and is not assigned a composite score, percentage, or confirmatory ranking.

Metabolomic analysis of the PMC10887286 dataset (plasma/urine, *n* = 336) showed exploratory enrichment of energy metabolism pathways. Glyoxylate and dicarboxylate metabolism reached FDR = 0.006, whereas the TCA cycle (FDR = 0.057) and pyruvate metabolism (FDR = 0.089) were suggestive and did not meet FDR < 0.05 (Figure 4C). Key differential metabolites included 2-ketoglutaric acid and nonanoic acid.

### 3.5. External Validation and Phenotypic Extension in FinnGen

To assess the transportability of the urate–kidney genetic relationship and to explore SERPINC1-associated phenotypes, we performed complementary analyses using FinnGen R12. These analyses are described as external validation or phenotypic extension, not as complete one-to-one replication of every CKDGen outcome.

**Figure 5:**
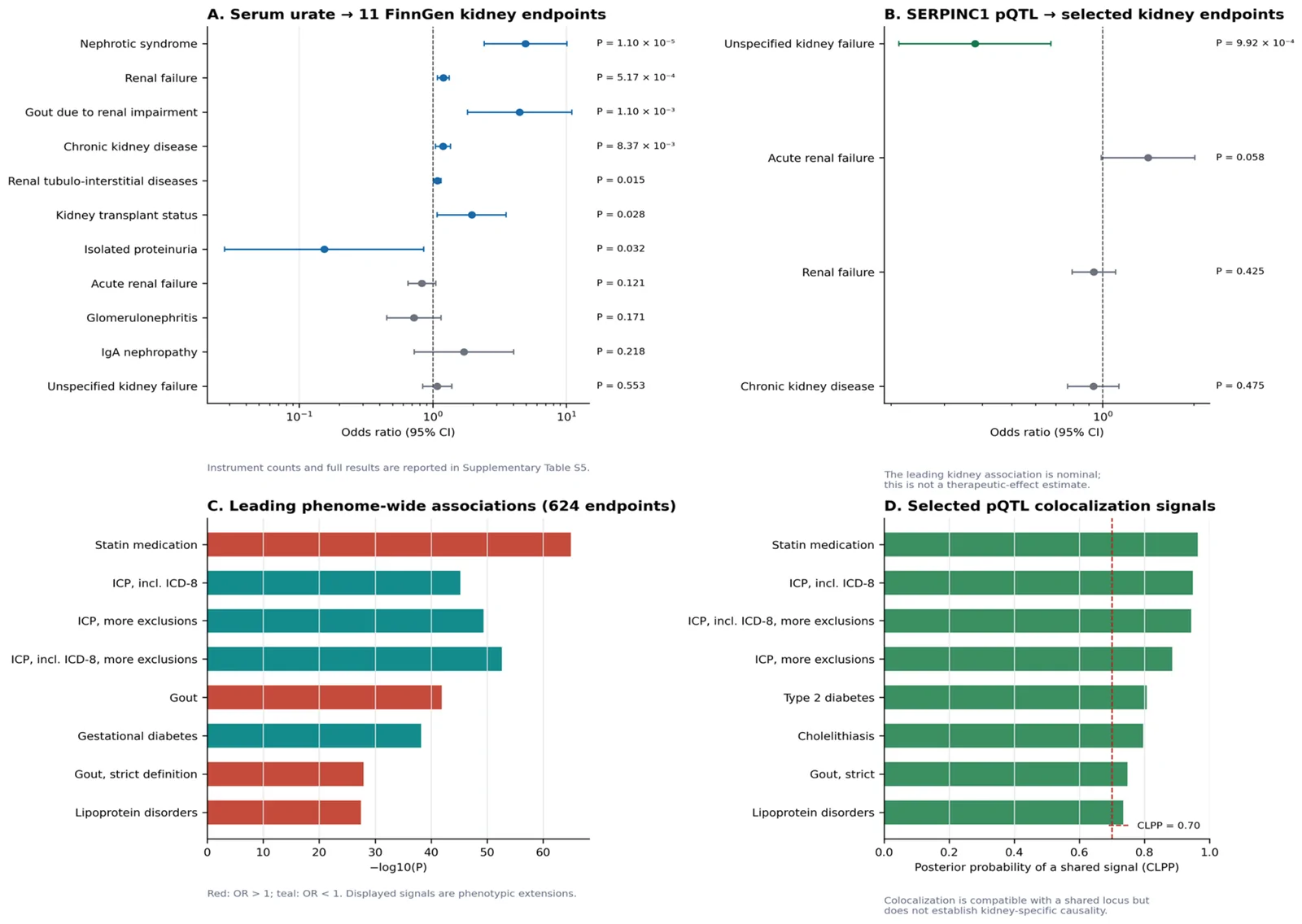
External validation and phenotypic extension in FinnGen R12. (**A**) Forest plot of serum urate associations with all 11 pre-defined kidney endpoints; points show odds ratios and horizontal lines show 95% confidence intervals. (**B**) SERPINC1 pQTL MR results across four selected kidney endpoints; the association with unspecified kidney failure was nominal (OR = 0.38, *p* = 9.9 × 10^−4^). (**C**) Leading phenome-wide associations from the 624-endpoint scan; bar length shows −log10(P), with red and teal indicating OR > 1 and OR < 1, respectively. (**D**) Selected pQTL-phenotype colocalization signals; bars show CLPP and the dashed line marks CLPP = 0.70. These analyses are external validation or phenotypic extension and do not establish kidney-specific causality.

External validation of the urate-to-kidney MR. The original 351 urate GWAS IVs were mapped to 11 pre-defined FinnGen kidney endpoints. Associations were reported for nephrotic syndrome (OR = 4.95, 95% CI: 2.43–10.10, *p* = 1.10 × 10^−5^), renal failure (OR = 1.20, 95% CI: 1.08–1.33, *p* = 5.2 × 10^−4^), gout due to impaired renal function (OR = 4.47, 95% CI: 1.82–10.99, *p* = 1.1 × 10^−3^), and chronic kidney disease (OR = 1.19, 95% CI: 1.05–1.36, *p* = 8.4 × 10^−3^) (Figure 5A, Supplementary Table S5). The nephrotic-syndrome *p* value is taken from the submitted table row and is consistent with the reported OR and confidence interval.

SERPINC1 pQTL MR in kidney endpoints. Using the submitted UK Biobank PPP pQTL instruments, we observed a nominal association with unspecified kidney failure (OR = 0.38, *p* = 9.9 × 10^−4^), but no significant effects on acute renal failure, renal failure overall, or chronic kidney disease (Figure 5B, Supplementary Table S3). These protein-level results require cautious interpretation because of instrument number and pleiotropy.

Phenome-wide MR across 624 FinnGen R12 endpoints. The strongest reported SERPINC1 pQTL signals were concentrated in metabolic traits, gout, type 2 diabetes, and lipoprotein disorders, intrahepatic cholestasis of pregnancy, and coagulation-related phenotypes rather than kidney endpoints (Figure 5C, Supplementary Table S2). Bayesian colocalization showed high posterior support for shared signals with selected metabolic and gout traits, but this pattern is compatible with pleiotropy and does not establish a kidney-specific effect. The originating FinnGen endpoint metadata and quantified participant-overlap estimate were not recoverable from the supplied archive.

A representative scatter plot for the nephrotic syndrome endpoint visually demonstrates the consistency of the urate-to-kidney association across individual IVs (Supplementary Figure S5A). Finally, an integrative framework diagram summarizes the multi-layer evidence from SERPINC1 genetic loci, cis-eQTL MR, pQTL MR, colocalization, PheWAS, and FinnGen validation (Supplementary Figure S5B).

### 3.6. Exploratory Multi-Omics Evidence Summary

**Figure 6:**
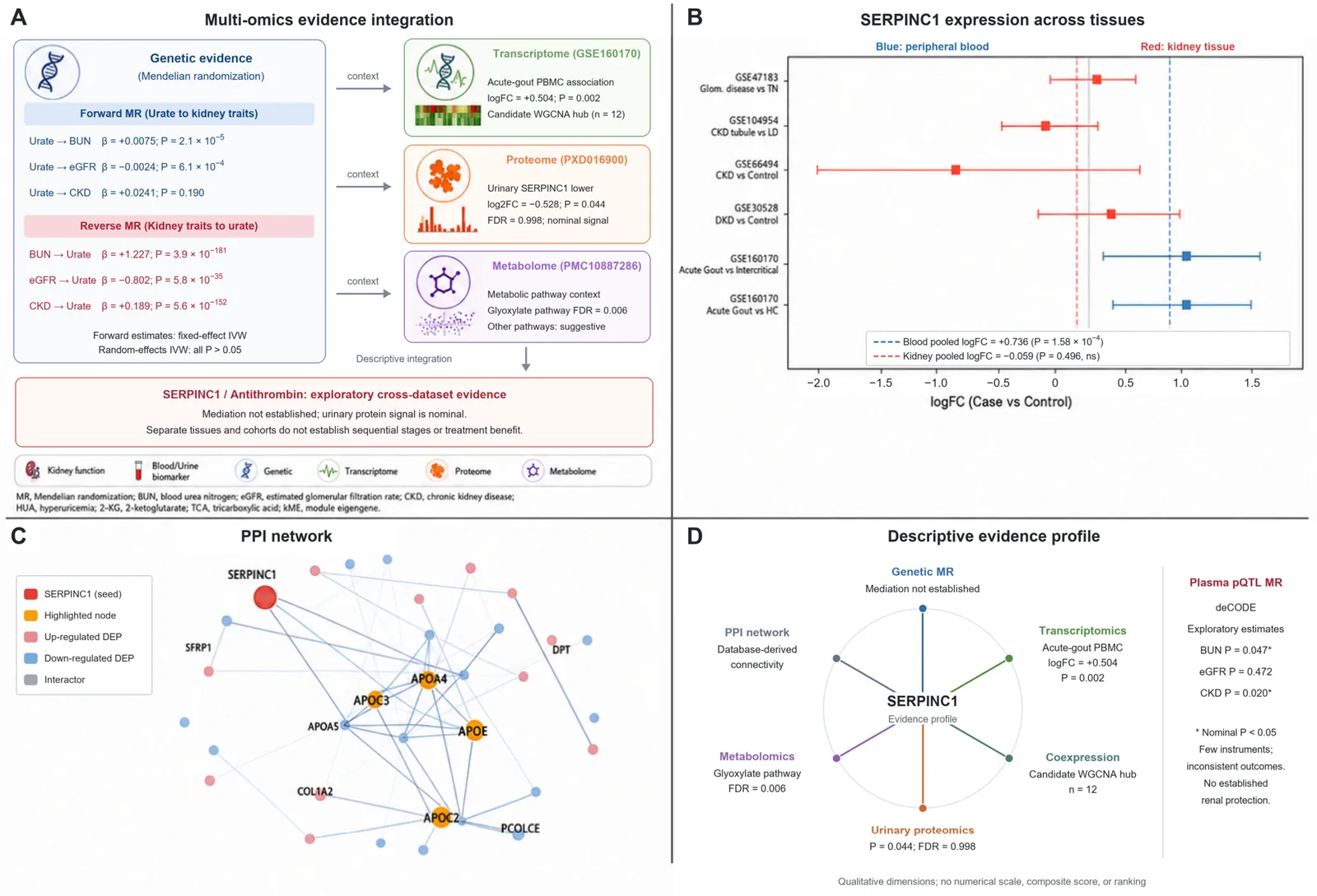
Exploratory multi-omics evidence map for SERPINC1. (**A**) Descriptive evidence map showing urate-kidney estimates, reciprocal reverse-direction estimates, the nominal urinary SERPINC1 difference, and higher PBMC SERPINC1 transcription during acute gout. Tissue, cohort, and platform differences preclude interpretation as a proven sequential mechanism. (**B**) SERPINC1 transcriptomic comparisons in peripheral blood (blue; two contrasts from GSE160170) and kidney tissue (red; four datasets). Squares indicate estimates, horizontal bars indicate confidence intervals, and dashed lines indicate tissue-group pooled estimates; ns, not significant. (**C**) Database-derived PPI network. Larger nodes visually highlight SERPINC1 and selected connected proteins; node size is not a measure of causal importance. Colors identify the SERPINC1 seed node, highlighted nodes, up- and down-regulated differentially expressed proteins (DEPs), and interactors. (**D**) Unscaled radial summary of six descriptive evidence dimensions: genetic MR, transcriptomic differential expression, exploratory coexpression, urinary proteomics, metabolomics, and PPI connectivity. Spoke lengths do not encode evidence strength. The adjacent plasma pQTL summary is exploratory; asterisks denote nominal significance (* *p* < 0.05). Across all dimensions, no composite score, percentage, or independent ranking claim is assigned.

Integrating the six evidence dimensions—MR genetic association, transcriptomic differential expression, exploratory coexpression, proteomic differential abundance, metabolomic pathway enrichment, and PPI network centrality—we constructed an exploratory model (Figure 6A). The model presents stage-associated and cross-dataset patterns; it does not assert two sequential biological stages.

**Chronic hyperuricemic dataset.** This dataset showed a nominal urinary SERPINC1 difference, whereas the acute-gout PBMC dataset showed higher SERPINC1 transcription. Because these observations came from different cohorts, tissues or biofluids, and platforms, they are presented as a stage-associated cross-dataset pattern rather than evidence of consumptive depletion or a validated sequential process.

**Acute-gout PBMC dataset.** Metabolomic pathway findings provide exploratory metabolic context. Glyoxylate and dicarboxylate metabolism reached FDR = 0.006, whereas the TCA cycle and pyruvate metabolism had FDR = 0.057 and 0.089, respectively, and did not meet the conventional FDR < 0.05 threshold. These data did not directly measure SERPINC1 or antithrombin and therefore cannot establish a direct SERPINC1 metabolic mechanism.

A summary display visualizes SERPINC1 transcriptomic comparisons in peripheral blood and kidney tissue (Figure 6B). The PPI network summarizes database-derived connectivity (Figure 6C). The six evidence dimensions are shown descriptively in an unscaled radial layout (Figure 6D); no composite score, percentage, or confirmatory ranking is assigned.

## 4. Discussion

This study characterizes antithrombin (SERPINC1) as a responsive molecular feature in hyperuricemic nephropathy, while the available genetic data did not demonstrate mediation through genetically predicted whole-blood SERPINC1 expression. Higher PBMC expression during acute gout and a nominal urinary proteomic difference were observed in separate datasets. These findings are stage-associated cross-dataset patterns, not a validated biphasic or sequential mechanism.

Our MR analyses showed directionally consistent fixed-effect associations of genetically predicted serum urate with higher BUN and lower eGFR, but heterogeneity was substantial and the corresponding multiplicative random-effects estimates were not statistically significant. Leave-one-out analyses retained the direction of the full estimate, although this diagnostic does not resolve biological pathway heterogeneity or residual pleiotropy. Reverse-direction estimates were also associated with serum urate. Because BUN, eGFR, and CKD are related but non-identical traits with different scales, these results support reciprocal genetic relationships rather than a proven temporal biological feedback process. This interpretation is consistent with prior clinical discussions of urate-related kidney injury [20]. In FinnGen R12, selected kidney endpoints provided external validation and phenotypic extension; the nephrotic-syndrome estimate was OR = 4.95 (95% CI: 2.43–10.10, *p* = 1.10 × 10^−5^).

The available SERPINC1 analyses did not demonstrate mediation through genetically predicted whole-blood expression. The planned two-step estimate was not estimable because the required first step was not adequately supported, and the eQTL results cannot exclude kidney-specific or non-genetic mediation. The complementary pQTL analysis used only three common instruments and produced inconsistent renal estimates: nominally lower BUN, null eGFR, and higher CKD risk with directional pleiotropy evidence. These findings should therefore be regarded as preliminary and do not support a general protective effect, antithrombin supplementation, or a therapeutic claim.

The multi-omics data showed higher SERPINC1 transcription in acute-gout PBMCs and a nominal urinary proteomic difference in a separate hyperuricemia cohort. The urinary result did not survive multiple-testing correction (FDR = 0.998), and the small PBMC dataset contained only 12 subjects. Accordingly, these findings are best treated as exploratory, stage-associated signals requiring validation in larger, matched cohorts [21,22,23]. Their different tissues, fluids, cohorts, and platforms prevent inference of sequential phases or consumptive depletion.

The metabolomic findings provide context for metabolic dysregulation but do not directly link SERPINC1 to the affected pathways. Glyoxylate and dicarboxylate metabolism met FDR < 0.05, whereas the TCA cycle and pyruvate metabolism did not. These results support biological plausibility for a metabolic–thromboinflammatory context [24,25], but they cannot substitute for a direct molecular or mediation analysis of SERPINC1.

The translational implications should remain hypothesis-generating. Urinary antithrombin and PBMC SERPINC1 expression may warrant evaluation as candidate measurements in future studies, but their clinical utility has not been established. The nominal BUN pQTL association is inconsistent with the null eGFR and directionally discordant CKD results, and the CKD analysis showed directional pleiotropy. Therefore, the current data do not justify claims of a renal-protective treatment or antithrombin supplementation [26]. Future work should use larger, tissue-matched cohorts, complete FinnGen metadata, and prespecified validation analyses.

This study has several limitations. First, the urate–kidney MR analyses showed high heterogeneity, and the multiplicative random-effects estimates were not statistically significant for the main outcomes; residual horizontal pleiotropy cannot be excluded. Second, participant overlap between the urate GWAS and kidney outcome datasets, including FinnGen, could not be quantified from the available materials. Third, SERPINC1 eQTLs were derived from whole blood and may not represent kidney-specific regulation. Fourth, GSE160170 contains only 12 subjects, with three in each gout subgroup, making coexpression topology and connectivity estimates potentially unstable. Fifth, the urinary SERPINC1 result was nominal (*p* = 0.044) and did not survive FDR correction (FDR = 0.998). Sixth, the pQTL analysis used only three common cis instruments, showed inconsistent kidney outcomes, and included a CKD directional-pleiotropy signal. Seventh, the metabolomic analysis did not directly measure SERPINC1, and the TCA and pyruvate findings did not meet FDR < 0.05. Finally, complete FinnGen endpoint definitions and case/control counts were not recoverable, and the multi-omics dimensions have not been validated as a quantitative target-prioritization score.

## 5. Conclusions

This integrated analysis found directionally consistent fixed-effect genetic associations of serum urate with renal traits, alongside reciprocal reverse-direction associations. Because of substantial heterogeneity and different trait scales, these results should be interpreted as reciprocal genetic relationships rather than a proven biological feedback cycle. Available data did not demonstrate mediation through genetically predicted whole-blood SERPINC1 expression. Higher PBMC expression during acute gout and a nominal urinary proteomic difference were observed in separate datasets and should be treated as exploratory, stage-associated patterns. The pQTL findings were preliminary and inconsistent and do not establish a modifiable therapeutic target.

## Figures and Tables

**Table 1:** Summary of baseline characteristics of public datasets used in this study.

Dataset	Purpose of Analysis	Sample Size	Study Population/Subgroups	Omics Type	Testing Platform	References	Population
GCST90319904	MR exposure variable (uric acid instrumental variable)	*n* ≈ 288,649 (European population)	Serum uric acid levels (continuous)	Genetic GWAS	Genome-wide association study	GWAS Catalog GCST90319904; Cho 2024 [11]	European
CKDGen BUN	MR Outcome—Blood Urea Nitrogen	*n* ≈ 210,000	BUN (continuous)	Genetic GWAS	Genome-wide association study	Wuttke 2019 [12]	European
CKDGen eGFR	MR Outcome —Glomerular Filtration Rate	*n* ≈ 520,000	eGFR (continuous)	Genetic GWAS	Genome-wide association study	Wuttke 2019 [12]	European
CKDGen CKD	MR Outcome—Chronic Kidney Disease	406,000 cases	CKD (binary)	Genetic GWAS	Genome-wide association study	Wuttke 2019 [12]	European
GSE160170	SERPINC1 transcriptomic differential expression	*n* = 12 (3–6 cases per group)	Acute gout/interval/control	Transcriptome	Microarray GPL21827	Qing 2021 [13]	Human PBMC
PXD016900	Quantification of the proteome in hyperuricemic urine	*n* = 51 (HUA 26 vs. Ctrl 25)	Hyperuricemia/healthy controls	Proteomics	LC-MS/MS MaxQuant	Huo 2021 [14]	Human Urine
PMC10887286	TCA cycle and metabolic pathway analysis	*n* = 336 (Gout group vs. AHU group)	Gout/asymptomatic Hyperuricemia	Metabolomics	LC-MS Targeted/Non-targeted	Ohashi 2024 [15]	Human Plasma/Urine
eQTLGen Phase 1	SERPINC1 eQTL MR instrumental variables	*n* = 31,684 (peripheral blood)	Whole blood cis-eQTL	eQTL	RNA-seq whole blood	Võsa 2021 [16]	European
deCODE SomaScan pQTL	SERPINC1 pQTL MR instrumental variables	*n* = 35,559	Plasma protein levels (continuous)	pQTL	SomaScan v4	Ferkingstad 2021 [17]	European
UKB PPP Olink 3k	SERPINC1 pQTL MR instruments	*n* = ~3000	Plasma protein levels (continuous)	pQTL	Olink Proximity Extension Assay	Sun 2023 [18]	European
FinnGen R12	External validation of urate–kidney MR and PheWAS	*n* = ~377,277	624 disease endpoints	GWAS	Illumina + Affymetrix	Kurki 2023 [19]	European

Note: BUN = blood urea nitrogen; eGFR = estimated glomerular filtration rate; CKD = chronic kidney disease; HUA = hyperuricemia; AHU = asymptomatic hyperuricemia; MR = Mendelian randomisation; GWAS = genome-wide association study. PBMC = Peripheral Blood Mononuclear Cell. All datasets are publicly available and do not involve individual patient privacy.

**Table 2:** Instrumental variables for SERPINC1 cis-eQTL.

SNP	Position	Beta	SE	*p*-Value	F	EA
rs9425780	chr1:173903300	0.2108	0.0060	1.9 × 10^−266^	1216	A
rs12564992	chr1:174478100	0.1933	0.0060	2.3 × 10^−230^	1050	G
rs41266044	chr1:174221460	0.1799	0.0057	3.0 × 10^−219^	999	C
rs61828068	chr1:174731998	0.1736	0.0057	1.6 × 10^−203^	927	A
rs12561796	chr1:173633528	0.1504	0.0057	9.0 × 10^−154^	698	T
rs12140932	chr1:173370335	0.0649	0.0057	7.9 × 10^−30^	129	A
rs60503324	chr1:173015598	0.0320	0.0057	2.0 × 10^−8^	32	A

**Table 3:** Mendelian Randomization Analysis Results of Uric Acid on Renal Function.

Outcome	N_IV	Method	Beta	SE	*p*-Value	Q (df)	I^2^	Egger P
BUN	331	Fixed-effect IVW	+0.00745	0.00175	2.05 × 10^−5^	2852 (330)	88.4%	0.280
BUN	328	MR-PRESSO	+0.00818	0.00177	3.56 × 10^−6^	—	—	—
BUN	331	Random-effects IVW	+0.00745	0.00515	0.147	—	—	—
eGFR	341	Fixed-effect IVW	−0.00235	0.00069	6.13 × 10^−4^	7227 (340)	95.3%	0.872
eGFR	338	MR-PRESSO	−0.00295	0.00069	1.80 × 10^−5^	—	—	—
eGFR	341	Random-effects IVW	−0.00235	0.00316	0.457	—	—	—
CKD	343	Fixed-effect IVW	+0.02410	0.01840	0.190	1800 (342)	81.0%	0.660
CKD	340	MR-PRESSO	+0.03567	0.01848	0.054	—	—	—
CKD	343	Random-effects IVW	+0.02410	0.04221	0.568	—	—	—

**Table 4:** Bidirectional Mendelian Randomization Analysis Results.

Causal Direction	N_IV	Beta	SE	*p*-Value
Urate → BUN	331	+0.00745	0.00175	2.05 × 10^−5^
Urate → eGFR	341	−0.00235	0.00069	6.13 × 10^−4^
Urate → CKD	343	+0.02410	0.01840	0.190
BUN → Urate	54	+1.227	0.043	3.9 × 10^−181^
eGFR → Urate	119	−0.802	0.065	5.8 × 10^−35^
CKD → Urate	20	+0.189	0.007	5.6 × 10^−152^

**Table 5:** Mendelian Randomization Analysis Results of SERPINC1 eQTL.

Outcome	N_IV	Beta	SE	*p*-Value	Q P	Egger P
BUN	7	−0.00334	0.00334	0.317	0.867	0.228
eGFR	7	+0.00180	0.00131	0.171	0.972	0.140
CKD	7	+0.07537	0.03562	0.034	0.992	0.790

**Table 6:** Mendelian Randomization Analysis Results of SERPINC1 pQTL.

Outcome	N_IV	Beta	SE	*p*-Value	Q P	Egger P
BUN	3	−0.0290	0.0146	0.047	0.996	0.781
eGFR	3	−0.0036	0.0049	0.472	0.728	—
CKD	3	+0.3361	0.1442	0.020	0.289	0.028

## Data Availability

All datasets used in this study were obtained from publicly available databases or published studies. The serum urate GWAS instruments were obtained from the GWAS Catalog study GCST90319904 [11] (https://www.ebi.ac.uk/gwas/studies/GCST90319904; accessed on 7 September 2026). Summary statistics for BUN, eGFR, and CKD were obtained from the CKDGen Consortium dataset [12] (https://ckdgen.imbi.uni-freiburg.de/datasets/archive/2019/; accessed on 7 September 2026). GSE160170 was downloaded from GEO [13]. The urinary proteomic dataset PXD016900 was obtained from ProteomeXchange/PRIDE [14]. Metabolomics data were extracted from Ohashi et al. [15] and its Supplementary Materials. SERPINC1 cis-eQTL summary data were obtained from eQTLGen Phase 1 [16], and cis-pQTL instruments were derived from the deCODE plasma proteome GWAS [17]. FinnGen pQTL analyses used UKB-PPP Olink 3k data [18] and FinnGen R12 summary data [19]. The FinnGen analyses are reported as external validation and phenotypic extension. Endpoint-level metadata and quantified participant overlap were not available in the supplied archive. The PPI network was constructed using STRING. Analyzed result tables and scripts are available from the corresponding author upon reasonable request.

## Supplementary Materials

The following supporting information can be downloaded at: https://www.mdpi.com/article/10.3390/genes17091084/s1: Figure S1: Study Design and Analytical Workflow; Figure S2: Sensitivity analyses for urate-to-kidney MR (A, MR-PRESSO; B, funnel plots; C, multi-method analysis); Figure S3: SERPINC1 eQTL MR sensitivity analyses (A, leave-one-out analyses; B, funnel plots); Figure S4: WGCNA soft-threshold selection and cross-dataset SERPINC1 expression meta-analysis; Figure S5: FinnGen urate-to-nephrotic-syndrome scatter plot and SERPINC1 multi-layer framework; Table S1: deCODE SERPINC1 cis-pQTL instruments; Table S2: FinnGen PheWAS results; Table S3: SERPINC1 pQTL MR for selected FinnGen kidney endpoints; Table S4: pQTL-disease colocalization results; Table S5: serum urate MR for 11 FinnGen kidney endpoints; Table S6: descriptive multi-omics evidence dimensions.

## Abbreviations

AT	Antithrombin
BUN	Blood Urea Nitrogen
CKD	Chronic Kidney Disease
eGFR	estimated Glomerular Filtration Rate
eQTL	expression quantitative trait locus
FDR	false discovery rate
GWAS	genome-wide association study
UAN	hyperuricemic nephropathy
IVW	inverse-variance weighted
IVs	Instrumental variables
MR	Mendelian Randomization
PBMC	Peripheral Blood Mononuclear Cell
PheWAS	phenome-wide association scan
PPI	Protein–Protein Interaction
SNPs	single-nucleotide polymorphisms
TCA cycle	Tricarboxylic Acid Cycle
WGCNA	Weighted Gene Co-expression Network Analysis
