# Supplementary material for: Serum Urate and Renal Dysfunction: Bidirectional Genetic Associations, External Validation, and Exploratory Multi-Omics Characterization of Antithrombin (SERPINC1) in Hyperuricemic Nephropathy
Source: Genes (Basel). 2026 Sep 9;17(9):1084. doi: 10.3390/genes17091084 (PMC13606670; doi:10.3390/genes17091084)
Supplement: Supplementary file 1 [file genes-17-01084-s001.zip › genes-4527282-supplementary.pdf]

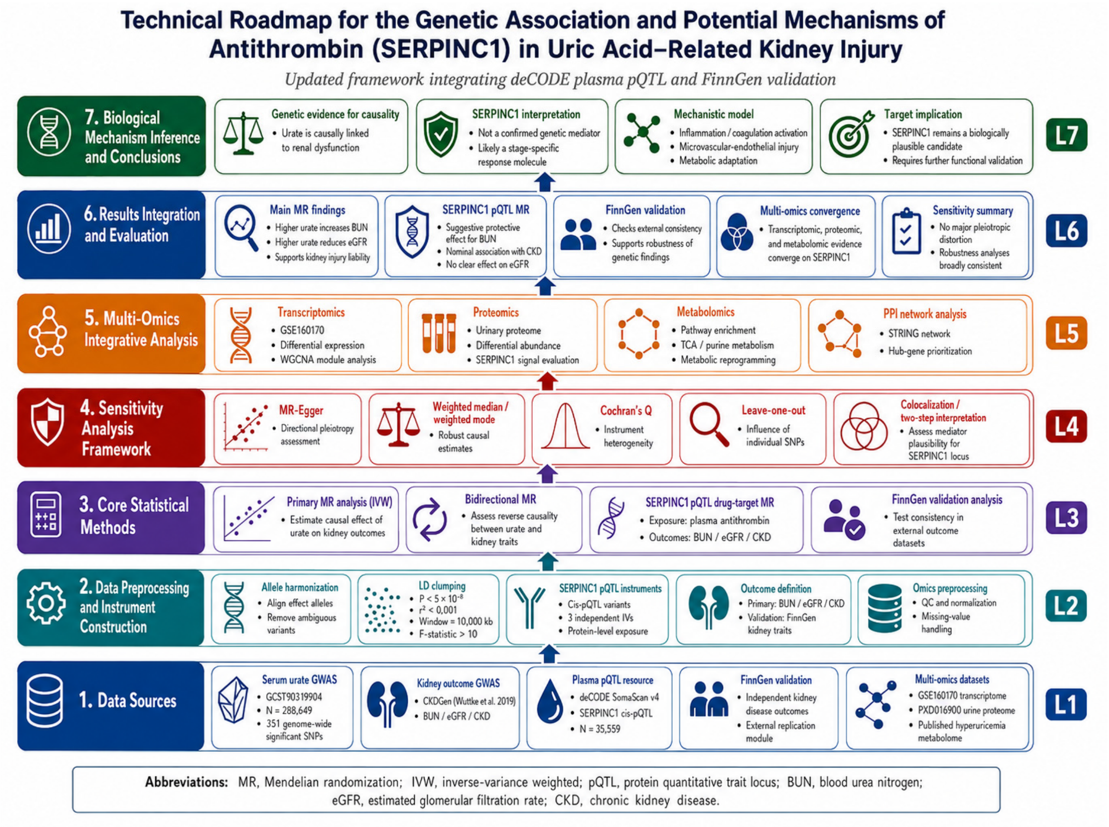

Supplementary Figure S1. Study Design and Analytical Workflow. The framework is structured into seven hierarchical levels (L1–L7), beginning with data infrastructure (genomic, transcriptomic, proteomic, metabolomic, and clinical data) and progressing through data harmonization, core statistical methods (MR, colocalization), sensitivity analyses, multi-omics integration, and finally biological mechanism evaluation and functional validation. Abbreviations: MR, Mendelian randomization; eQTL, expression quantitative trait locus; pQTL, protein quantitative trait locus; WGCNA, weighted gene co-expression network analysis; PPI, protein-protein interaction; PheWAS, phenome-wide association study.

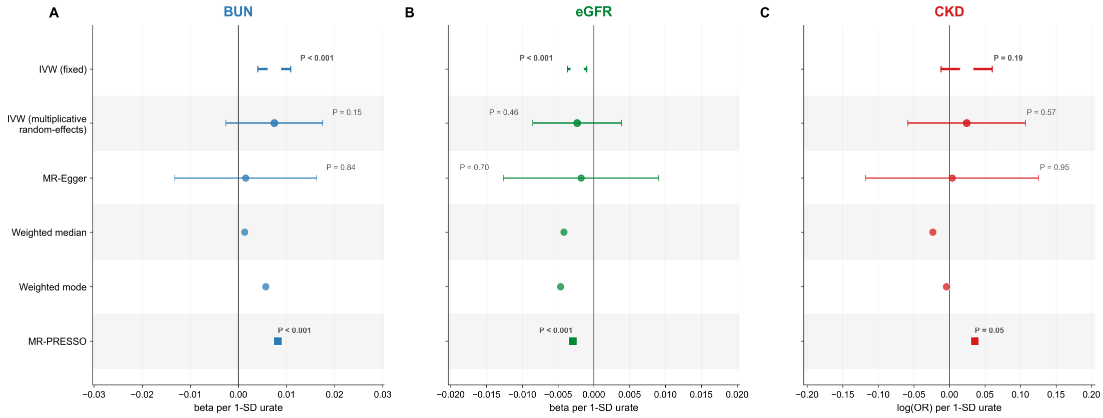

Sensitivity analysis summary

	Cochran Q	df	I^2	Egger intercept	Egger P	MR-PRESSO
BUN	2852.3	330	88.4%	0.00028	0.280	3 outliers, P=3.56e-06
eGFR	7227.5	340	95.3%	-2.52e-05	0.872	3 outliers, P=1.80e-05
CKD	1800.2	342	81.0%	0.00092	0.660	3 outliers, P=0.054

Exposure: GCST90319904 (serum urate, N=288,649). Outcomes: CKDGen 2019 (Wuttke et al., Nat Genet). Error bars: 95% CI.

Supplementary Figure S2A. MR-PRESSO outlier-corrected Mendelian randomization estimates for urate-to-kidney outcomes. As a sensitivity analysis, removing three outlier variants per outcome left directions unchanged for BUN ($P = 3.56 \times 10^{-6}$), eGFR ($P = 1.80 \times 10^{-5}$), and CKD ($P = 0.054$); this does not by itself establish robustness. Error bars represent 95% confidence intervals.

Funnel-plot sensitivity analyses for serum urate to kidney outcomes

Distribution of single-SNP Wald ratios around the overall IVW estimate

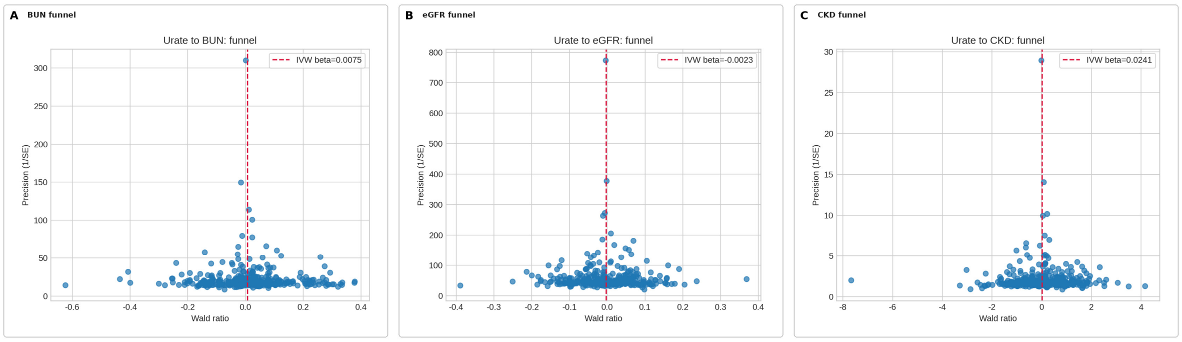

Supplementary Figure S2B. Funnel plots for urate-to-kidney Mendelian randomization. Panels A, B, and C correspond to BUN, eGFR, and CKD outcomes, respectively. Each dot represents a single instrumental variable Wald ratio estimate. No clear systematic funnel-plot asymmetry was observed, although residual pleiotropy cannot be excluded.

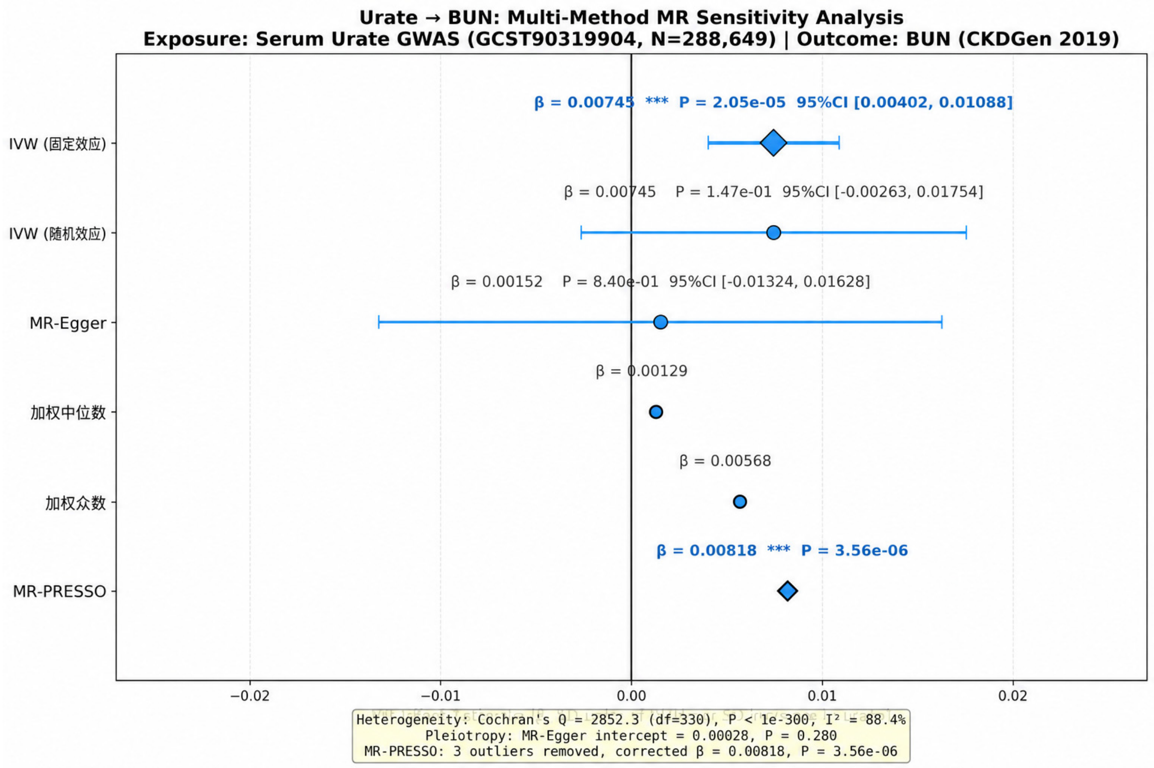

Supplementary Figure S2C. Multi-method MR sensitivity analysis forest plot for the urate-to-BUN association. Comparison of fixed-effects IVW, multiplicative random-effects IVW, MR-Egger, weighted median, weighted mode, and MR-PRESSO estimates. Directions were similar; random-effects IVW was not significant, whereas MR-PRESSO remained significant.

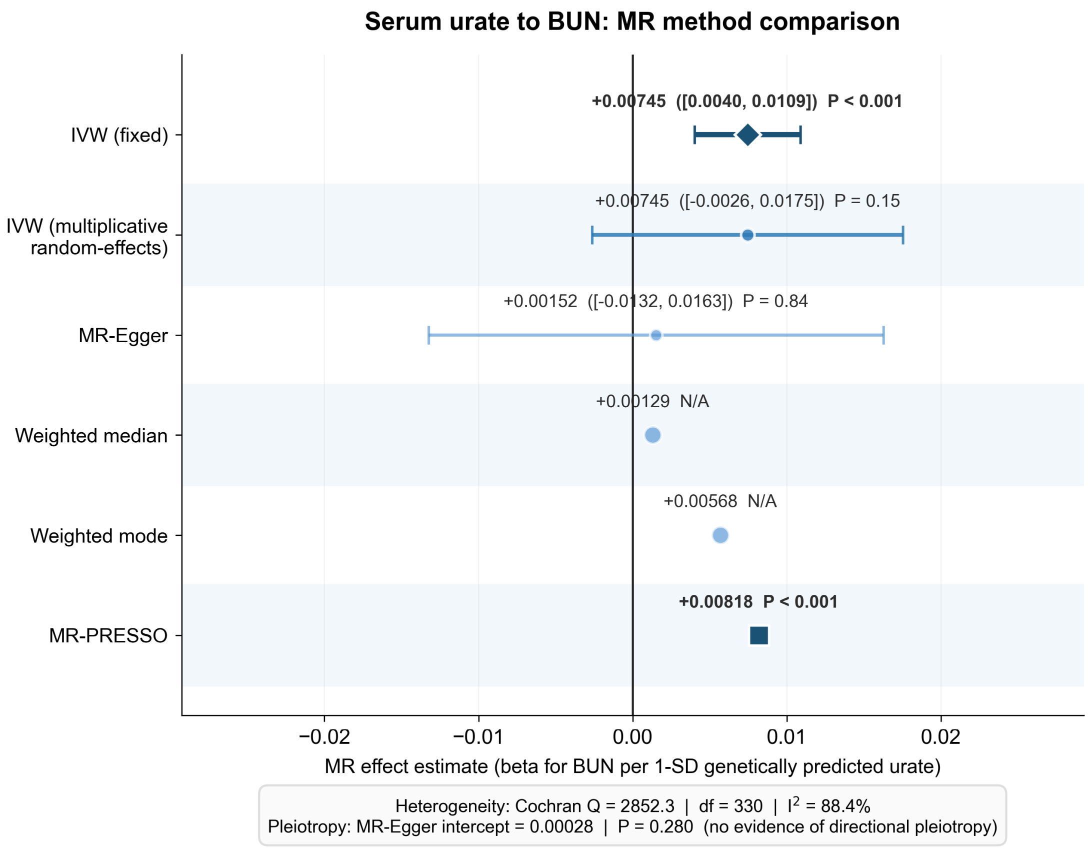

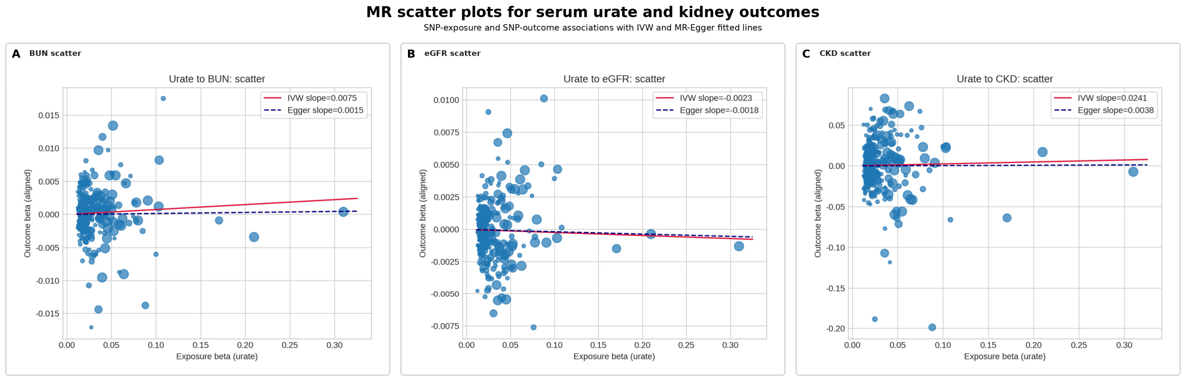

Supplementary Figure S3. SERPINC1 eQTL MR sensitivity analyses. (A) Leave-one-out analyses for BUN, eGFR, and CKD. (B) Funnel plots for BUN, eGFR, and CKD. No single variant clearly dominated the estimates; residual pleiotropy cannot be excluded.

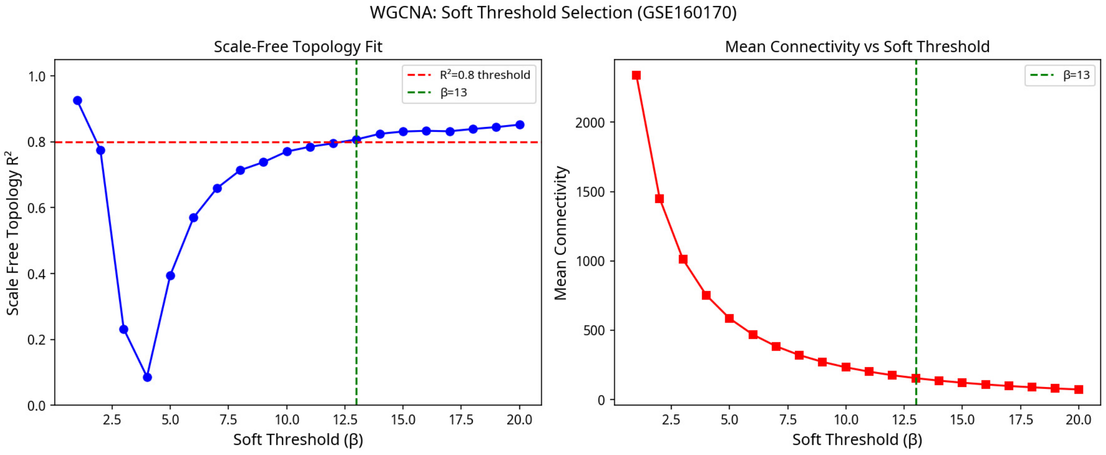

Supplementary Figure S4A. Determination of Soft Threshold Power for WGCNA Network Construction. The plot shows the soft thresholding analysis for the weighted gene co-expression network. The x-axis represents the soft thresholding power (β). The left y-axis shows the scale-free topology model fit (signed R^2), and the right y-axis shows the mean connectivity. At β = 13, the signed R^2 was 0.807; this value was used for subsequent WGCNA analysis. This value (β=13) was chosen as the optimal soft thresholding power for the subsequent WGCNA module identification analysis.

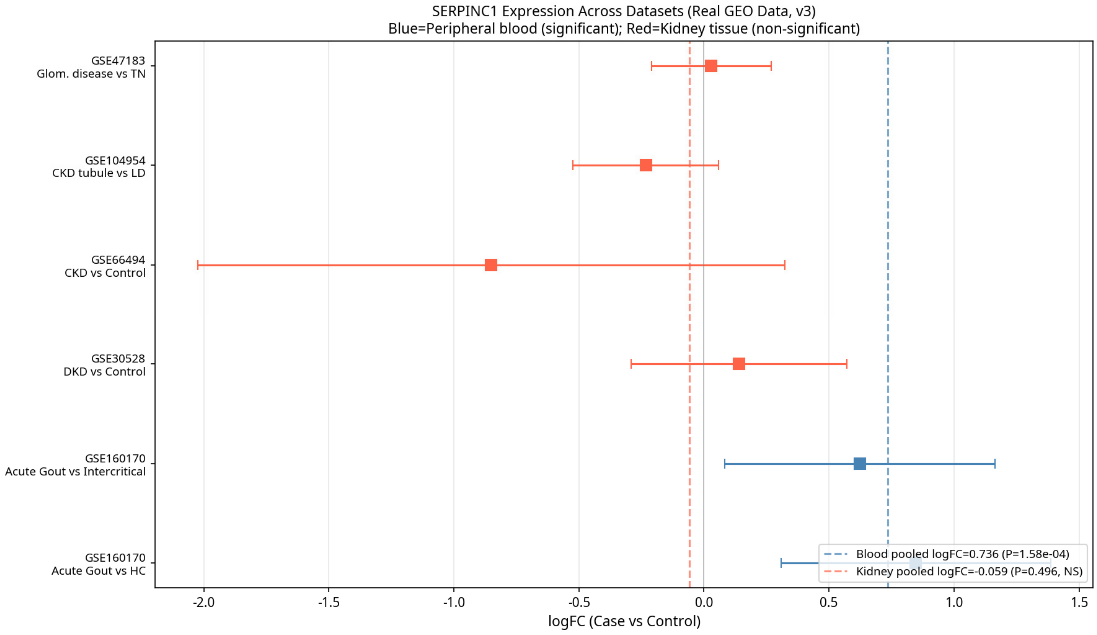

Supplementary Figure S4B. Cross-dataset meta-analysis forest plot of SERPINC1 expression. Peripheral blood datasets showed consistent SERPINC1 upregulation (pooled logFC = +0.736, P = 1.58×10^{-4}, I^2 = 0%), while renal tissue datasets showed no differential expression (pooled logFC = −0.059, P = 0.496). This pattern is tissue-specific but exploratory.

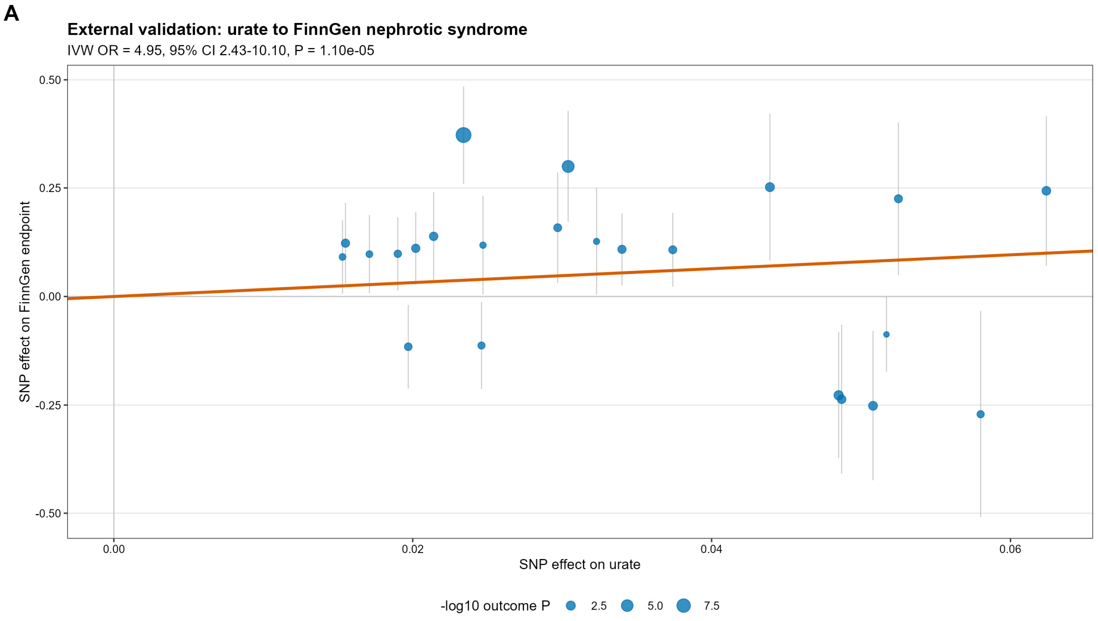

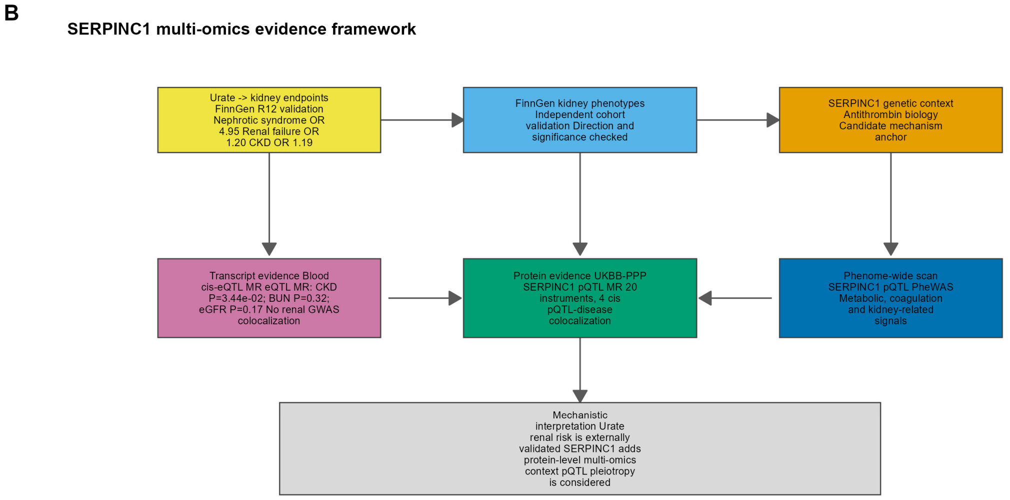

Supplementary Figure S5A. Representative scatter plot for urate-to-nephrotic syndrome external validation (FinnGen R12). Each dot represents a single urate instrumental variable. The x-axis shows the SNP effect on serum urate; the y-axis shows the SNP effect on nephrotic syndrome. The IVW line is overlaid (OR = 4.95, 95% CI: 2.43–10.10, P = 1.10 × 10^{-5}).

Supplementary Figure S5B. Integrative framework diagram of SERPINC1 multi-layer evidence. The diagram summarizes SERPINC1 loci, whole-blood cis-eQTL MR, plasma pQTL MR, pQTL-disease colocalization, PheWAS, and FinnGen kidney external validation. It is an exploratory framework and does not establish mediation, sequential stages, or therapeutic effects.

Supplementary Table S1. cis-pQTL instrumental variables for SERPINC1 (deCODE plasma proteome). List of four independent cis-pQTL SNPs within ±1 Mb of the *SERPINC1* gene from deCODE SomaScan v4 (N = 35,559 Icelandic/European). Columns: SNP ID, pQTL P-value, F-statistic, MAF, effect allele (EA), GWAS matching status (BUN/eGFR/CKD), and notes. Three variants (rs116185683, rs141846315, rs112808303) were used as the primary pQTL MR instrumental variables across all three kidney outcomes; rs2016525 was available only for CKD.

SNP	pQTL P-value	F-statistic	MAF	EA	GWAS Matching	Notes
rs116185683	2.31e-18	76	0.010	T	BUN/eGFR/CKD	Primary IV
rs141846315	1.38e-07	28	0.032	T	BUN/eGFR/CKD	Primary IV
rs2016525	6.81e-06	20	0.042	TG	CKD	Outcome-specific candidate
rs112808303	7.50e-06	20	0.042	A	BUN/eGFR/CKD	Primary IV

Supplementary Table S2. Top phenome-wide association scan (PheWAS) results for SERPINC1 pQTL in FinnGen R12. List of top FinnGen phenotypes associated with genetically predicted SERPINC1 protein levels, including phenocode, phenotype description, phenotypic group, number of instrumental variables (N_inst), odds ratio (OR), lower and upper 95% confidence intervals (CI_low, CI_high), P-value, and FDR-adjusted P-value. Bonferroni-significant endpoints (N = 141) and FDR-significant endpoints (N = 393) were identified across 624 scanned endpoints. The strongest signals cluster in metabolic traits (statin medication, gout, type 2 diabetes, lipoprotein disorders), intrahepatic cholestasis of pregnancy, and coagulation-related phenotypes.

phenocode	phenotype	group	n_inst	OR	CI_low	CI_high	p	FDR
RX_STATIN	Statin medication	Metabolic	8	1.6167	1.5302	1.7081	1.09e-65	6.79e-63
O15_ICP_WIDE_EXMORE	Intrahepatic Cholestasis of Pregnancy (ICP) , incl. ICD-8 (more control exclusions)	Pregnancy	5	0.0781	0.0564	0.1081	2.33e-53	7.26e-51
O15_ICP_EXMORE	Intrahepatic Cholestasis of Pregnancy (ICP) (more control exclusions)	Pregnancy	5	0.0657	0.0459	0.0941	4.60e-50	9.56e-48
O15_ICP_WIDE	Intrahepatic Cholestasis of Pregnancy (ICP) , incl. ICD-8	Pregnancy	6	0.1058	0.0777	0.1442	5.84e-46	9.11e-44
M13_GOUT	Gout	Metabolic	6	3.0713	2.6151	3.6070	1.36e-42	1.70e-40
GEST_DIABETES	Gestational diabetes (for exclusion)	Metabolic	3	0.3322	0.2815	0.3919	6.00e-39	6.24e-37
GOUT_STRICT	Gout, strict definition	Metabolic	6	3.2783	2.6585	4.0425	1.18e-28	1.05e-26
E4_LIPOPROT	Disorders of lipoprotein metabolism and other lipidaemias	Metabolic	4	1.6441	1.5049	1.7962	3.32e-28	2.59e-26
E4_HYPERLIPMIX	Mixed hyperlipidaemia	Metabolic	3	16.8259	10.1187	27.9789	1.43e-27	9.94e-26
GOUT_NOS	Gout, unspecified	Metabolic	6	2.8932	2.3800	3.5172	1.53e-26	9.57e-25
T2D_WIDE	Type 2 diabetes, wide definition	Metabolic	5	0.6221	0.5690	0.6801	1.78e-25	1.01e-23
E4_HYPERCHOL	Pure hypercholesterolaemia	Metabolic	3	1.7786	1.5922	1.9869	2.13e-24	1.11e-22

Supplementary Table S3. Mendelian randomization results of SERPINC1 pQTL for selected FinnGen kidney endpoints. Results are shown for unspecified kidney failure, acute renal failure, renal failure, and chronic kidney disease. Columns include phenocode, phenotype, analysis, instrument count, OR, 95% CI, P-value, and FDR. The unspecified-kidney-failure association was nominal (OR = 0.3785, P = 9.92 × 10^{-4}).

phenocode	phenotype	analysis	n_inst	OR	CI_low	CI_high	p	FDR
N14_RENFAILNAS	Unspecified kidney failure	SERPINC1 pQTL all genome-wide instruments	2	0.3785	0.2123	0.6748	9.92e-04	2.61e-03
N14_ACUTERENFAIL	Acute renal failure	SERPINC1 pQTL all genome-wide instruments	2	1.4116	0.9884	2.0159	5.80e-02	8.78e-02
N14_RENFAIL	Renal failure	SERPINC1 pQTL all genome-wide instruments	3	0.9351	0.7930	1.1026	4.25e-01	5.19e-01
N14_CHRONKIDNEYDIS	Chronic kidney disease	SERPINC1 pQTL all genome-wide instruments	3	0.9310	0.7650	1.1330	4.75e-01	5.63e-01

Supplementary Table S4. Bayesian colocalization results between SERPINC1 pQTL and FinnGen disease traits. Colocalization analysis results from FinnGen R12 gene pQTL colocalization API (source: UK Biobank PPP

Olink 3k). Columns: phenotype, phenotype description, lead pQTL variant (pqtl_variant), posterior probability of shared causal variant (CLPP), posterior probability of distinct causal variant (CLPA), disease GWAS P-value (disease_sep), and pQTL P-value (pqtl_P). High colocalization support (CLPP > 0.7) was observed for statin medication, intrahepatic cholestasis of pregnancy, type 2 diabetes, gout, and lipoprotein metabolism disorders.

phenotype	phenotype_description	pqtl_variant	CLPP	CLPA	disease_p	pqtl_p
RX_STATIN	Statin medication	2:27508073:T:C	0.9639	0.9653	6.24e-36	3.24e-33
O15_ICP_WIDE	Intrahepatic Cholestasis of Pregnancy (ICP), incl. ICD-8	2:27508073:T:C	0.9487	0.9653	4.07e-26	3.24e-33
O15_ICP_WIDE_EXMORE	Intrahepatic Cholestasis of Pregnancy (ICP), incl. ICD-8 (more control exclusions)	2:27508073:T:C	0.9434	0.9653	1.85e-25	3.24e-33
O15_ICP_EXMORE	Intrahepatic Cholestasis of Pregnancy (ICP) (more control exclusions)	2:27508073:T:C	0.8848	0.9165	4.59e-24	3.24e-33
T2D	Type 2 diabetes, definitions combined	2:27508073:T:C	0.8066	0.8354	4.16e-19	3.24e-33
K11_CHOLELITH	Cholelithiasis	3:172576710:G:A	0.7954	0.7964	2.13e-08	3.98e-11
GOUT_STRICT	Gout, strict definition	2:27508073:T:C	0.7476	0.7744	7.69e-14	3.24e-33
E4_LIPOPROT	Disorders of lipoprotein metabolism and other lipidaemias	2:27508073:T:C	0.7344	0.7607	2.61e-16	3.24e-33
M13_GOUT	Gout	2:27508073:T:C	0.6704	0.6943	1.62e-20	3.24e-33
K11_CHOLECYSTECTOMY	Cholecystectomy	2:27508073:T:C	0.6157	0.6377	4.93e-21	3.24e-33
KELA_DIAB_INSUL_EXMORE	Diabetes, insulin treatment (Kela reimbursement) (more control exclusions)	2:27508073:T:C	0.5806	0.6016	2.87e-17	3.24e-33
K11_CHOLELITH	Cholelithiasis	2:27508073:T:C	0.5659	0.5864	2.66e-20	3.24e-33

Supplementary Table S5. Mendelian randomization results of serum urate for 11 FinnGen kidney endpoints. The original 351 urate instruments were mapped to 11 pre-defined endpoints. Columns include phenocode,

phenotype, instrument count, OR, 95% CI, P-value, and FDR. Reported associations included nephrotic syndrome (OR = 4.9489, P = 1.10 $\times 10^{-5}$), renal failure (P = 5.17 $\times 10^{-4}$), gout due to impaired renal function (P = 1.10 $\times 10^{-3}$), and chronic kidney disease (P = 8.37 $\times 10^{-3}$).

phenocode	phenotype	n_inst	OR	CI_low	CI_high	p	FDR
N14_NEPHROTICSYND	Nephrotic syndrome	23	4.9489	2.4257	10.0968	1.10e-05	1.21e-04
N14_RENFAIL	Renal failure	54	1.1987	1.0821	1.3279	5.17e-04	2.84e-03
GOUT_KIDNEY	Gout due to impairment of renal function	21	4.4710	1.8187	10.9910	1.10e-03	4.04e-03
N14_CHRONKIDNEYDIS	Chronic kidney disease	51	1.1930	1.0463	1.3603	8.37e-03	2.30e-02
N14_RENALTUB	Renal tubulo-interstitial diseases	50	1.0823	1.0154	1.1537	1.51e-02	3.32e-02
Z21_KIDNEY_TRANSPLANT_STATUS	Kidney transplant status	24	1.9541	1.0758	3.5496	2.78e-02	5.10e-02
N14_ISOLPROTEINURIA	Isolated proteinuria with specified morphological lesion	16	0.1538	0.0276	0.8550	3.24e-02	5.10e-02
N14_ACUTERENFAIL	Acute renal failure	35	0.8276	0.6517	1.0511	1.21e-01	1.66e-01
N14_GLOMER_NEPHRITIS	Glomerulonephritis	24	0.7223	0.4536	1.1503	1.71e-01	2.09e-01
N14_IGA_NEPHROPATHY	IgA nephropathy (IgA glomerulonephritis)	33	1.7126	0.7277	4.0307	2.18e-01	2.40e-01
N14_RENFAILNAS	Unspecified kidney failure	30	1.0788	0.8396	1.3862	5.53e-01	5.53e-01

Supplementary Table S6. Descriptive multi-omics evidence dimensions for SERPINC1. Descriptive evidence dimensions for SERPINC1: (1) MR genetic association (urate→kidney), (2) transcriptomic differential expression (acute gout vs control, GSE160170), (3) WGCNA module membership (kME = 0.953, blue module), (4)

proteomic differential abundance (hyperuricemia vs control, PXD016900), (5) metabolomic pathway enrichment (FDR < 0.05, PMC10887286), and (6) PPI network centrality (degree = 50, STRING). No composite score, percentile, or ranking is reported; the dimensions are descriptive and not independent. Note: This table corresponds to the descriptive evidence display in Figure 6D; no numerical score is assigned.

**Evidence Dimension**	**Status**	**Criteria**
1. MR genetic association (urate→kidney)	-	Descriptive evidence only; no composite score.
2. Transcriptomic differential expression (acute gout vs control)	-	Differential signal described; no composite score.
3. WGCNA module membership (kME)	-	Connectivity described; no composite score.
4. Proteomic differential abundance (hyperuricemia vs control)	-	Abundance described; no composite score.
5. Metabolomic pathway enrichment (FDR < 0.05)	-	Pathways described; no composite score.
6. PPI network centrality (hub degree)	-	Centrality described; no hub score.
Summary	--	No score
